# Upregulated of ANXA3, SORL1, and Neutrophils May Be Key Factors in the Progressionof Ankylosing Spondylitis

**DOI:** 10.3389/fimmu.2022.861459

**Published:** 2022-04-06

**Authors:** Jie Jiang, Xinli Zhan, Haishun Qu, Tuo Liang, Hao Li, Liyi Chen, Shengsheng Huang, Xuhua Sun, Wenyong Jiang, Jiarui Chen, Tianyou Chen, Yuanlin Yao, Shaofeng Wu, Jichong Zhu, Chong Liu

**Affiliations:** ^1^ Department of Spinal Orthopedic Surgery, The First Clinical Affiliated Hospital of Guangxi Medical University, Nanning, China; ^2^ Department of Traditional Chinese Medicine, The People’s Hospital of Guangxi Zhuang Autonmous Region, Nanning, China

**Keywords:** Ankylosing spondylitis, immune cell infiltration, immune cell correlation analysis, hypoxia, high-throughput sequencing, immunohistochemical analysis, BASDAI

## Abstract

**Introduction:**

The specific pathogenesis of ankylosing spondylitis (AS) remains unclear, and our study aimed to investigate the possible pathogenesis of AS.

**Materials and Methods:**

Two datasets were downloaded from the GEO database to perform differentially expressed gene analysis, GO enrichment analysis, KEGG pathway analysis, DO enrichment analysis, GSEA analysis of differentially expressed genes, and construction of diagnostic genes using SVM and WGCNA along with Hypoxia-related genes. Also, drug sensitivity analysis was performed on diagnostic genes. To identify the differentially expressed immune genes in the AS and control groups, we analyzed the composition of immune cells between them. Then, we examined differentially expressed genes in three AS interspinous ligament specimens and three Degenerative lumbar spine specimens using high-throughput sequencing while the immune cells were examined using the neutrophil count data from routine blood tests of 1770 HLA-B27-positive samples and 7939 HLA-B27-negative samples. To assess the relationship between ANXA3 and SORL1 and disease activity, we took the neutrophil counts of the first 50 patients with above-average BASDAI scores and the last 50 patients with below-average BASDAI scores for statistical analysis. We used immunohistochemistry to verify the expression of ANXA3 and SORL1 in AS and in controls.

**Results:**

ANXA3 and SORL1 were identified as new diagnostic genes for AS. These two genes showed a significant differential expression between AS and controls, along with showing a significant positive correlation with the neutrophil count. The results of high-throughput sequencing verified that these two gene deletions were indeed differentially expressed in AS versus controls. Data from a total of 9707 routine blood tests showed that the neutrophil count was significantly higher in AS patients than in controls (p < 0.001). Patients with AS with a high BASDAI score had a much higher neutrophil count than those with a low score, and the difference was statistically significant (p < 0.001). The results of immunohistochemistry showed that the expression of ANXA3 and SORL1 in AS was significantly higher than that in the control group.

**Conclusion:**

Upregulated of ANXA3, SORL1, and neutrophils may be a key factor in the progression of Ankylosing spondylitis.

## Introduction

Ankylosing spondylitis (AS) is a chronic inflammatory disease whose cause is not fully understood (1, 2). Compared to other inflammatory diseases, the innate immune system plays a dominant role in AS. Additionally, biomechanical factors (e.g., periosteal microdamage) may also play an important role in the pathogenesis of joint manifestations of AS with the immune cells in the periosteum having a link to genetic susceptibility, chronic inflammation, and local damage (3). More than half the population with AS is male, along with genetic susceptibility in more than 90%. The strongest genetic link of AS is with HLA-B27 (4). AS can lead to the development of spinal ankylosis and cause structural damage, which is irreversible (5). The pathological changes and osteoimmunological features of AS mainly include osteolytic bone destruction and osteogenic changes. The interaction of T cells, B cells, osteoblasts, and osteoclasts together regulate the pathogenesis of AS (6). More importantly, the long course of AS as a chronic inflammatory disease makes the emotional and financial devastation of the patient unbearable. Thus, there is an urgent need to find more accurate biomarkers for pre-diagnosis and possible pathogenesis of AS.

Immune cell infiltration plays an important role in inflammatory diseases. Kidney damage has been leading to chronic inflammation, causing an imbalance between infiltrating immune cells and resident cells (7). Furthermore, in human autoimmune systemic diseases, the persistence of an activated autoreactive B-cell response, triggered by the persistence of autoreactive B cells, is considered a key factor in the development of rheumatoid arthritis (8). The T-cell profile is determined by the differential expression and specific functions of the cytokines, surface antigens, and transcription factors, while the defects in unconventional T cells are particularly relevant to autoimmunity and chronic inflammation (9). However, studies are also available on immune cells involved in diseases that appear to be very precisely articulated not just in rheumatoid arthritis and chronic inflammatory nephrotic injury but also in many other diseases (10–12). Interestingly, research is still insufficient on this aspect of AS, where specific mechanisms need further elucidation for AS as an immune-mediated and continuously progressive disease.

Hypoxia is an important feature of the immune ecology of human physiology and pathology, and the effects of hypoxia on immunity and inflammation may vary depending on the immune processes and microenvironment occurring in specific ecological niches (13). In rheumatoid arthritis, the highly dysregulated structure of the microvascular system results in inadequate synovial oxygenation, which continues to increase with the metabolic turnover of the expanding synovial vascular cataract, resulting in a hypoxic microenvironment (14). Osteoarthritis (OA), a common aging-related disease, is caused by an imbalance between extracellular matrix degradation and and formation, and an abnormal chondrocyte metabolism in response to changes in the inflammatory microenvironment may play a key role in cartilage degeneration and OA progression; under environmental stress conditions, chondrocytes may adapt to changes in the microenvironment by altering metabolic pathways, which may be oxidative phosphorylation to glycolysis (15, 16). However, the specific mechanisms by which hypoxia plays a role in the development of ankylosing spondylitis, a chronic inflammatory disease, remain unclear, and there is little research on hypoxia in AS.

Our research aimed to explore the genes closely related to Hypoxia and immunity in AS using a bioinformatics approach along with searching new diagnostic genes for AS and the impact of immune cells on it. It is necessary to investigate in depth the important role assumed by hypoxia genes in AS and the relationship with the interaction with immune cells.

## Materials and Methods

### Data Download and Processing

We downloaded two AS datasets from the Gene Expression Omnibus (GEO) repository for analyzing the possible diagnostic markers. GSE25101 (17) (https://www.ncbi.nlm.nih.gov/geo/query/acc.cgi?acc=GSE25101) dataset contained a total of 32 samples while the GSE73754 (18) (https://www.ncbi.nlm.nih.gov/geo/query/acc.cgi) dataset contained a total of 72 samples. We first matched the probe names of the two datasets to their corresponding gene symbols using the programming language PERL (v5.30.2) and then, de-batched and normalized the two datasets so that they could be analyzed at the same level. We combined the AS samples from both datasets and the control group into a single file for unified analysis. In this study, we used the turning language PERL for text processing and turning language R (version 4.0.2) for statistical analysis and drawing of images.

### Differential Expression Analysis and Construction of Heat and Volcano Plots

To analyze the differentially expressed genes between the AS and control groups, we used the ‘limma’ package to carry out an in-depth analysis between the two groups. The cut-off values were set to |logFC|filter = 0.2 and adjusted to p < 0.05. The details of differentially expressed genes are given in **Table 1**. We also used the ‘pheatmap’ package to visualize differentially expressed genes as heat maps, while the ‘dplyr’ package, ‘ggplot2’ package, and ‘ggrepel’ package were used to visualize the genes with significant differential expression shown in the volcano maps.

**Table 1:** List of differentially expressed genes in the GEO dataset.

id	logFC	AveExpr	t	P.Value	adj.P.Val	B
ANXA3	1.885866	5.997883	1.515039	0.18595	0.687908	-4.57257
GTSF1	-2.29224	3.501843	-7.7654	0.000398	0.687908	-4.46981
ERVMER34-1	1.75741	3.322894	7.5776	0.00045	0.687908	-4.47044
SIGLEC7	-3.03417	3.956009	-7.5284	0.000465	0.687908	-4.47062
SIRPB2	-2.07544	4.6068	-7.24593	0.000563	0.687908	-4.47167
OLIG1	-3.20651	4.22382	-6.81295	0.000763	0.687908	-4.47352
ZNF671	-3.22314	4.334445	-6.54864	0.000925	0.687908	-4.4748
TRAT1	-1.97044	4.424755	-6.42015	0.001019	0.687908	-4.47547
CFAP53	1.475623	3.094979	6.320359	0.001099	0.687908	-4.47602
CD300LF	-2.39527	4.35351	-6.13666	0.001268	0.687908	-4.47708
WDR5B	1.364584	3.01688	6.115105	0.001289	0.687908	-4.47721
ZNF610	1.905029	3.38294	6.077071	0.001328	0.687908	-4.47745
OGDHL	-3.04014	4.522351	-5.92583	0.001499	0.687908	-4.47841
GPC6	-2.36038	3.5975	-5.90556	0.001523	0.687908	-4.47854
PAX3	3.449629	4.079747	5.740176	0.001744	0.687908	-4.47967
ZNF257	1.361445	3.146668	5.723928	0.001767	0.687908	-4.47979
PI15	-3.55252	4.27259	-5.56726	0.002014	0.687908	-4.48094
SKIDA1	1.498002	3.257387	5.542464	0.002057	0.687908	-4.48113
ABCA5	1.927374	3.879314	5.444391	0.002237	0.687908	-4.4819
ADGRE1	-2.64619	4.443619	-5.34293	0.002442	0.687908	-4.48274
CCK	1.908288	3.663797	5.292791	0.002551	0.687908	-4.48316
PLCB2	-2.67209	8.182263	-5.28764	0.002563	0.687908	-4.48321
STON1-GTF2A1L	1.951009	3.407893	5.284031	0.002571	0.687908	-4.48324
DOCK5	-2.13725	4.903934	-5.27083	0.002601	0.687908	-4.48335
ZNF215	-3.06059	4.222684	-5.16113	0.002866	0.687908	-4.48433
PLCD3	1.63188	3.433442	5.154728	0.002883	0.687908	-4.48439
ANKRD13C	1.774652	3.422037	5.122087	0.002968	0.687908	-4.48469
ANK3	1.622336	3.125966	5.116879	0.002982	0.687908	-4.48474
PAMR1	2.503824	10.37158	5.083202	0.003074	0.687908	-4.48505
ITK	-2.556	4.349861	-5.07639	0.003093	0.687908	-4.48512
LIMD2	-1.9764	3.868977	-5.0486	0.003171	0.687908	-4.48538
HIST1H3H	-1.68422	3.398773	-5.04277	0.003188	0.687908	-4.48544
CDO1	2.434362	12.78293	5.03086	0.003223	0.687908	-4.48555
MED12L	1.902582	4.968718	5.011924	0.003279	0.687908	-4.48573
PTCRA	-3.06644	4.886228	-5.00027	0.003314	0.687908	-4.48585
RTN4R	-2.05557	4.980805	-4.98998	0.003345	0.687908	-4.48595
IL2RG	-1.35695	3.211495	-4.98458	0.003362	0.687908	-4.486
CCL28	1.732048	5.066258	4.965683	0.003421	0.687908	-4.48619
CATSPER3	1.464888	3.197241	4.965168	0.003422	0.687908	-4.4862
NUMB	-1.27642	6.071224	-4.91503	0.003584	0.687908	-4.4867
MT1X	3.319116	10.81005	4.87171	0.003731	0.687908	-4.48715
ZNF493	1.216413	4.53603	4.819048	0.003918	0.687908	-4.4877
SLA	-1.76912	3.590584	-4.81189	0.003945	0.687908	-4.48778
XRCC6BP1	1.287327	6.632254	4.791808	0.00402	0.687908	-4.48799
ATG12	1.614728	3.756053	4.787384	0.004037	0.687908	-4.48804
PNPLA5	1.344145	3.14481	4.775056	0.004084	0.687908	-4.48817
CD300A	-1.65026	4.954222	-4.75678	0.004155	0.687908	-4.48837
SPOPL	1.117646	2.860095	4.755062	0.004161	0.687908	-4.48839
PTPRH	3.058408	4.487313	4.741081	0.004217	0.687908	-4.48854
LYZ	-3.00502	10.20791	-4.73373	0.004246	0.687908	-4.48863
RSPO4	-1.61407	3.290838	-4.69586	0.004402	0.687908	-4.48905
MYL3	5.649118	7.162862	4.692241	0.004417	0.687908	-4.48909
LANCL3	-2.29394	4.096554	-4.68294	0.004456	0.687908	-4.4892
PCDH8	1.985251	3.710278	4.650695	0.004596	0.687908	-4.48956
SORL1	-0.41137	2.611965	-0.9253	0.394298	0.700408	-4.59882
TBX5	1.998665	3.426011	4.640922	0.004639	0.687908	-4.48968
TYROBP	-2.18624	11.21439	-4.63907	0.004648	0.687908	-4.4897
FOXF2	2.195612	3.817484	4.627773	0.004698	0.687908	-4.48983
EVI2B	-2.32889	7.465002	-4.61537	0.004755	0.687908	-4.48997
ZNF98	1.177059	2.964217	4.612958	0.004766	0.687908	-4.49
CEACAM21	-1.37212	3.549147	-4.58927	0.004876	0.687908	-4.49028
PTTG1	-2.48442	5.494549	-4.58174	0.004912	0.687908	-4.49037
LINC01272	-3.25805	7.151908	-4.57107	0.004963	0.687908	-4.4905
SHANK2	-3.04636	4.433093	-4.54255	0.005102	0.687908	-4.49084
LYN	-1.50608	3.443499	-4.52848	0.005173	0.687908	-4.49101
ZNF717	2.029827	3.775699	4.490927	0.005366	0.687908	-4.49147
CTRB1	2.246512	3.899304	4.453171	0.005569	0.687908	-4.49195
PAH	-1.35373	6.538216	-4.44287	0.005626	0.687908	-4.49208
APOBEC3B	-1.34202	7.255544	-4.44152	0.005634	0.687908	-4.4921
FMOD	2.965108	12.27452	4.413575	0.005792	0.687908	-4.49245
SI	-3.03924	4.901569	-4.40239	0.005856	0.687908	-4.4926
FGF22	-1.83315	4.935606	-4.39505	0.005899	0.687908	-4.49269
LOC728485	-1.50648	4.950007	-4.38256	0.005973	0.687908	-4.49286
ZNF711	1.129725	2.944516	4.358532	0.006118	0.687908	-4.49317
TTC30A	1.496536	3.09189	4.306065	0.006449	0.687908	-4.49387
TSC22D1	1.071982	2.894408	4.26843	0.006698	0.687908	-4.49439
SLC1A4	1.415072	9.509514	4.264835	0.006723	0.687908	-4.49444
SH3BP1	-2.01441	7.775217	-4.25718	0.006775	0.687908	-4.49455
HIST2H3C	-4.2686	5.286366	-4.22824	0.006978	0.687908	-4.49495
WAS	-2.3156	7.532127	-4.21935	0.007041	0.687908	-4.49508
LILRA3	-2.69284	4.04187	-4.20722	0.007129	0.687908	-4.49525
NUP210	-2.26544	7.002256	-4.20328	0.007158	0.687908	-4.49531
FAM178A	-2.59892	12.78206	-4.19419	0.007224	0.687908	-4.49544
UBXN2A	1.069235	5.265853	4.145528	0.007595	0.687908	-4.49614
DIXDC1	1.316749	8.714173	4.13041	0.007714	0.687908	-4.49636
RMND5A	1.546646	4.811428	4.125876	0.00775	0.687908	-4.49643
TACC3	-3.51896	4.5754	-4.11799	0.007814	0.687908	-4.49655
BIN2	-2.23246	8.681158	-4.11723	0.00782	0.687908	-4.49656
MT1E	2.032737	9.912271	4.107282	0.007901	0.687908	-4.49671
HESX1	1.203168	3.220966	4.100397	0.007957	0.687908	-4.49681
TSSK3	1.109502	4.580149	4.09658	0.007989	0.687908	-4.49687
CYP26B1	1.985526	9.41124	4.096338	0.007991	0.687908	-4.49687
DRC7	-2.45471	4.330347	-4.09404	0.00801	0.687908	-4.49691
WFIKKN2	1.955196	4.125592	4.088462	0.008057	0.687908	-4.49699
NLRC4	-2.15532	4.862855	-4.08611	0.008076	0.687908	-4.49703
ZNF154	-1.64739	6.066477	-4.08205	0.008111	0.687908	-4.49709
PAX7	1.386526	3.049544	4.048754	0.008397	0.687908	-4.49759
DDA1	-1.96314	5.726899	-4.03989	0.008475	0.687908	-4.49773
FYB	-1.64114	7.678873	-4.03943	0.008479	0.687908	-4.49774

### GO Enrichment Analysis and KEGG Pathway Enrichment Analysis

To gain more insight into the molecular functions (MF), biological processes (BP), cellular composition (CC), and pathway enrichment of these differentially expressed genes, we performed GO enrichment and KEGG pathway analysis using the “clusterProfiler” package, “org.Hs.eg.db” package, “enrichplot” package, “ggplot2” package, and “GOplot” package. Here, the “enrichplot”, “ggplot2”, and “GOplot” packages were used for the GO enrichment and KEGG pathway enrichment analysis for differentially expressed genes. The cut-off values were set and adjusted to p < 0.05.

### Disease Ontology Enrichment Analysis (DO) and Gene Set Enrichment Analysis (GSEA)

The DO enrichment analysis is important for understanding complex pathogenesis, diagnosis, and early prevention of major diseases (19–21). We used the ‘clusterProfiler’ package, ‘org.Hs.eg.db’ package, ‘enrichplot’ package, ‘ggplot2’ package, ‘GSEABase’ package, and ‘DOSE’ package to perform DO enrichment analysis and visualize the results of all differentially expressed genes. The GSEA enrichment analysis is a method used for genome-wide expression profiling of microarray data that integrates prior knowledge of localization, function, and biological significance of genes, which is used to construct a database of tags to understand their centralized expression (22–24). We used the ‘limma’ package, ‘org.Hs.eg.db’ package, ‘clusterProfiler’ package, and ‘enrichplot’ package to enrich the genes and visualize the results.

### Weighted Gene Co-Expression Network Analysis (WGCNA), Support Vector Machine (SVM) Analysis, Hypoxia-Related Gene Construction Prognostic Models, and ROC Diagnostic Curves

WGCNA is a framework used for building and analyzing weighted gene coexpression networks. It is a very advanced bioinformatics analysis method that uses two-two variable correlation coefficients to study biological networks. It selects the ones that satisfy the scale-free network topology by calculating soft thresholds and also calculates the coefficient of dissimilarity of each node, thus, building hierarchical clustering trees and finally finding the target genes and gene networks (25–27). SVM analysis methods are a class of generalized linear classifiers that can perform binary classification of data in a supervised learning manner, where the decision convenience is the maximum margin plane that can be solved for the learned samples, i.e., the separation hyperplane solves the dataset that correctly divides it and also has the largest geometric separation (28–30). We downloaded Hypoxia-related genes from the GSEA database (http://www.gsea-msigdb.org/gsea/msigdb/search.jsp) for the in-depth analysis and used these two advanced bioinformatics analysis techniques with Hypoxia-associated genes to take intersections for subsequent analysis. To test the accuracy of the constructed diagnostic model, ROC diagnostic curves were constructed using the “pROC” package.

### Drug Sensitivity Analysis

In order to better treat AS, a chronic inflammatory disease, and develop more drugs to improve the condition, we have performed an in-depth analysis of the drug sensitivity of genes closely related to AS and antineoplastic drugs in an attempt to find new drugs for better treatment of it. We downloaded gene expression files and drug sensitivity files from the CellMiner database (version: 2021.1, database: 2.6) and then used the “impute” package, the “limma” package, the “ggplot2” package and the “ggpubr” package for drug sensitivity analysis of ANXA3 and SORL1 for antitumor drugs.

### CIBERSORT Immune Cell Composition Analysis, Differential Analysis, and Genetic Correlation Analysis

The CIBERSORT software was developed by extracting the expression of genes in the immune cells as a reference value, and then a linear model was used to predict the number of immune cells in the sample, while a permutation test was used to assess the significance of the results (31–33). We quantified the content of 22 immune cells and defined the total immune cell content of each sample as 100%. After assessing the significance using a permutation test, we used the “vioplot” package to visualize the immune cell differences between the AS and control samples as violin plots. The ‘limma’ package, ‘reshape2’ package, ‘ggpubr’ package, and ‘ggExtra’ package were used to correlate immune cells with the genes. Finally, we also Lollipop Charted the proportion of immune cells and their differences between the AS and control groups.

### RNA Extraction, High-Throughput Sequencing, and Routine Blood Tests

To test the accuracy of our bioinformatics analysis, we performed a high-throughput sequencing analysis of three AS and three lumbar degenerative intervertebral ligament cases from the First Clinical Affiliated Hospital of Guangxi Medical University. The samples were taken intraoperatively for pathological testing and were used as external validation. This study was approved by the ethics department of the First Clinical Affiliated Hospital of Guangxi Medical University and was also compliant with the Declaration of Helsinki requirements for the analysis of intervertebral ligaments in anonymous patients. Therefore, this study also requested an exemption from patient informed consent. Inclusion criteria for the AS group were as follows: 1. AS with a posterior convexity deformity requiring surgical correction; 2. Patients who were not recently treated with medication or other modalities. The exclusion criteria included combined rheumatic disease and or with other serious organic diseases. Inclusion criteria for the lumbar degeneration group were as follows: 1. Those diagnosed with lumbar disc herniation and had reached the surgical threshold; 2. Those with a mild or normal loss of intervertebral space height with no bone destruction. The detailed baseline information for all cases can be found in **Table 2**. We first extracted the total RNA using the mirVana™ miRNA Isolation kit and then tested the quality of total RNA. After several laboratory operations such as cRNA synthesis and labeling, hybridization and washing, and microarray scanning, we obtained all mRNA expressions between the AS and lumbar degeneration groups. We then used the “limma” package to analyze the differentially expressed genes with the cut-off value set as before, which was |logFC|filter = 0.2, adjusted to p < 0.05. Having obtained all the differentially expressed genes, we placed the details of externally validated differentially expressed genes in **Table 3**. On the other hand, we also collected data on neutrophils, hematocrit, red blood cells, and white blood cells from 1770 HLA-B27-positive cases and 7939 HLA-B27-negative cases obtained from the First Clinical Affiliated Hospital of Guangxi Medical University and also statistically analyzed the two groups using the two independent samples t-test in SPSS 25. Finally, we visualized them using the R software. We also collected the Bath Ankylosing Spondylitis Disease Activity Index (BASDAI) (34) from 199 AS cases to assess disease activity in AS patients. We statistically analyzed the neutrophil counts of the first 50 patients with above-average BASDAI scores with the neutrophil counts of the last 50 patients with below-average BASDAI scores. In addition, we performed a correlation analysis of data from 1717 cases with complete data on both neutrophil and CRP blood routine examinations.

**Table 2:** Baseline information of all cases by high-throughput sequencing.

Evaluation index	Experimental group			Control group		
	AS1	AS2	AS3	IDD1	IDD2	IDD3
Age	55	24	44	56	52	26
Gender	Male	Male	Male	Male	Male	Male
HLA-B27	+	+	+	–	–	–
Bilateral sacroiliac joint injuries	+	+	+	–	–	–
Spinal and sacroiliac joint fusion	+	+	+	–	–	–

**Table 3:** List of differentially expressed genes by high-throughput sequencing.

id	logFC	AveExpr	t	P.Value	adj.P.Val	B
GTSF1	-2.29291	3.50218	-7.77368	0.000396	0.687818	-4.46982
CSF1R	1.757314	3.322952	7.577209	0.00045	0.687818	-4.47049
SIGLEC7	-3.03391	3.955901	-7.53001	0.000465	0.687818	-4.47065
SIRPB2	-2.07598	4.606899	-7.24805	0.000562	0.687818	-4.47171
ANXA9	-2.6564	8.467317	-3.67198	0.012584	0.687818	-4.504
OLIG1	-3.2077	4.224272	-6.82555	0.000756	0.687818	-4.4735
PLXNB1	-3.2228	4.334261	-6.55163	0.000923	0.687818	-4.47483
USHBP1	-1.97068	4.424878	-6.42037	0.001019	0.687818	-4.47551
CFAP53	1.474962	3.094639	6.318274	0.001101	0.687818	-4.47607
TNFSF8	-2.39486	4.353307	-6.13559	0.001269	0.687818	-4.47713
LPIN3	1.364373	3.016771	6.113	0.001291	0.687818	-4.47727
ZNF610	1.904149	3.38269	6.075852	0.00133	0.687818	-4.47749
C18orf65	-3.04045	4.522493	-5.92715	0.001497	0.687818	-4.47844
PTH2	-2.36058	3.597625	-5.90081	0.001529	0.687818	-4.47861
PAX3	3.449773	4.079791	5.7391	0.001745	0.687818	-4.47972
CISD2	1.361218	3.146632	5.721586	0.001771	0.687818	-4.47984
PI15	-3.5527	4.27262	-5.56705	0.002015	0.687818	-4.48098
SKIDA1	1.497776	3.25713	5.540097	0.002061	0.687818	-4.48119
ABCA5	1.92704	3.879282	5.442311	0.002241	0.687818	-4.48196
ADGRE1	-2.64629	4.443635	-5.34494	0.002437	0.687818	-4.48276
PLCB2	-2.67192	8.182244	-5.28764	0.002562	0.687818	-4.48325
CCK	1.90695	3.663796	5.284297	0.00257	0.687818	-4.48328
HSPD1	1.950523	3.40768	5.282294	0.002575	0.687818	-4.48329
DOCK5	-2.13762	4.903924	-5.27248	0.002597	0.687818	-4.48338
ZNF215	-3.06016	4.222478	-5.16226	0.002863	0.687818	-4.48436
PLCD3	1.631858	3.43341	5.155945	0.002879	0.687818	-4.48441
ANKRD13C	1.774299	3.421887	5.120964	0.002971	0.687818	-4.48474
ANK3	1.62204	3.125818	5.115645	0.002985	0.687818	-4.48479
PAMR1	2.503984	10.37153	5.083498	0.003073	0.687818	-4.48509
SMPDL3B	-2.55551	4.349839	-5.07498	0.003097	0.687818	-4.48517
LIMD2	-1.97668	3.868978	-5.04479	0.003182	0.687818	-4.48545
HIST1H3H	-1.68505	3.399101	-5.03782	0.003202	0.687818	-4.48552
TNS1	2.434791	12.78271	5.031098	0.003222	0.687818	-4.48559
MED12L	1.901763	4.968802	5.009523	0.003286	0.687818	-4.4858
PTCRA	-3.06596	4.886058	-4.99816	0.00332	0.687818	-4.48591
GTSF1L	-2.05564	4.980962	-4.99158	0.00334	0.687818	-4.48597
MATR3	-1.35681	3.211526	-4.98235	0.003369	0.687818	-4.48606
CCL28	1.732028	5.066495	4.972533	0.003399	0.687818	-4.48616
RASSF5	1.464541	3.197302	4.962336	0.003431	0.687818	-4.48626
OR2J2	-1.27695	6.070947	-4.91143	0.003596	0.687818	-4.48677
CAB39	3.319498	10.80986	4.872955	0.003726	0.687818	-4.48717
SLA	-1.76894	3.590525	-4.81109	0.003948	0.687818	-4.48782
ZNF493	1.215527	4.536097	4.809258	0.003954	0.687818	-4.48784
XRCC6BP1	1.287643	6.632228	4.792842	0.004016	0.687818	-4.48802
ATG12	1.614246	3.756424	4.787168	0.004037	0.687818	-4.48808
PNPLA5	1.343846	3.144621	4.774337	0.004086	0.687818	-4.48822
CD300A	-1.65025	4.954045	-4.75551	0.004159	0.687818	-4.48842
SPOPL	1.117556	2.86005	4.753874	0.004166	0.687818	-4.48844
VAMP1	3.058062	4.487409	4.739987	0.004221	0.687818	-4.48859
LYZ	-3.00534	10.20789	-4.73488	0.004241	0.687818	-4.48865
RBKS	5.648751	7.163057	4.692424	0.004416	0.687818	-4.48913
SORL1	-0.41125	2.612006	-0.92499	0.394447	0.700293	-4.59883
RSPO4	-1.61449	3.291027	-4.69142	0.00442	0.687818	-4.48914
TIGIT	-2.29424	4.096695	-4.6818	0.004461	0.687818	-4.48925
PCDH8	1.98546	3.710413	4.652403	0.004588	0.687818	-4.48958
TBX5	1.998564	3.426083	4.640501	0.004641	0.687818	-4.48972
STON1-GTF2A1L	-2.18616	11.21443	-4.63998	0.004643	0.687818	-4.48972
FOXF2	2.195352	3.817321	4.625876	0.004707	0.687818	-4.48989
CATSPER3	-2.32823	7.46506	-4.61682	0.004748	0.687818	-4.48999
TCN1	1.176995	2.964156	4.611959	0.00477	0.687818	-4.49005
CEACAM21	-1.3724	3.549258	-4.59163	0.004865	0.687818	-4.49029
PTTG1	-2.48482	5.494486	-4.5828	0.004907	0.687818	-4.49039
IGDCC3	-3.25758	7.151525	-4.57208	0.004958	0.687818	-4.49052
PNPLA6	-3.04654	4.433108	-4.54219	0.005104	0.687818	-4.49088
LYN	-1.50625	3.443653	-4.52537	0.005188	0.687818	-4.49109
ZNF717	2.030024	3.7756	4.491845	0.005361	0.687818	-4.4915
CTRB1	2.246054	3.899314	4.452776	0.005571	0.687818	-4.49199
MAMLD1	-1.35327	6.538209	-4.44424	0.005618	0.687818	-4.4921
APOBEC3B	-1.3418	7.255355	-4.441	0.005636	0.687818	-4.49214
TEN1	2.964952	12.2746	4.4126	0.005797	0.687818	-4.4925
SI	-3.03942	4.901296	-4.40474	0.005843	0.687818	-4.4926
ID4	-1.83361	4.935476	-4.39764	0.005884	0.687818	-4.4927
LOC728485	-1.50665	4.950098	-4.38679	0.005948	0.687818	-4.49284
POTEI	1.129474	2.944396	4.357012	0.006127	0.687818	-4.49323
TTC30A	1.496362	3.09179	4.305794	0.00645	0.687818	-4.49391
SLC1A4	1.415801	9.509721	4.269408	0.006692	0.687818	-4.49441
TSC22D1	1.071814	2.894313	4.267299	0.006706	0.687818	-4.49444
SH3BP1	-2.01434	7.775138	-4.25767	0.006772	0.687818	-4.49457
UBC	-4.26817	5.286224	-4.22765	0.006982	0.687818	-4.49499
WAS	-2.31538	7.531962	-4.22146	0.007026	0.687818	-4.49508
MMP26	-2.6936	4.042184	-4.21047	0.007105	0.687818	-4.49524
NUP210	-2.26556	7.002265	-4.20404	0.007152	0.687818	-4.49533
STOX2	-2.59928	12.78188	-4.19469	0.007221	0.687818	-4.49546
ERO1L	1.068823	5.266309	4.142883	0.007615	0.687818	-4.49621
DIXDC1	1.318022	8.714325	4.130423	0.007714	0.687818	-4.4964
RMND5A	1.545573	4.811443	4.12232	0.007779	0.687818	-4.49652
TACC3	-3.51875	4.575243	-4.11937	0.007802	0.687818	-4.49656
LOH12CR1	-2.2318	8.681364	-4.1147	0.00784	0.687818	-4.49663
MT1E	2.032855	9.912008	4.106179	0.00791	0.687818	-4.49676
OR10G8	1.203064	3.220907	4.099757	0.007963	0.687818	-4.49685
ABRACL	1.109068	4.579697	4.097212	0.007984	0.687818	-4.49689
CYP26B1	1.985719	9.411245	4.095838	0.007995	0.687818	-4.49691
DRC7	-2.45457	4.33042	-4.09376	0.008012	0.687818	-4.49694
NLRC4	-2.15601	4.862882	-4.08932	0.008049	0.687818	-4.49701
ZNF154	-1.64749	6.065975	-4.08578	0.008079	0.687818	-4.49706
WFIKKN2	1.954213	4.125819	4.085003	0.008085	0.687818	-4.49708
PAX7	1.386559	3.049537	4.047887	0.008404	0.687818	-4.49764
DDA1	-1.96316	5.726425	-4.0427	0.00845	0.687818	-4.49772
ZMIZ2	-1.64083	7.679025	-4.039	0.008483	0.687818	-4.49778

### Immunohistochemical Analysis

Here, five cases of interspinous ligaments diagnosed as AS with kyphosis and resected during surgery at the First Clinical Affiliated Hospital of Guangxi Medical University were used as the experimental group, and three cases of interspinous ligaments diagnosed as spinal fracture and resected during surgery were used as the normal control group to examine the differences in the expression of ANXA3 and SORL1 between the AS and control groups. The baseline information table for cases used for immunohistochemistry can be found in **Table 4**. Antibodies specific to ANXA3 for specific staining were purchased from the Proteintech (https://www.ptgcn.com/products/ANXA3-Antibody-11804-1-AP.htm, Catalog number: 11804-1-AP), dilution ratio of 1:200. SORL1-specific antibody was purchased from the abcam company (https://www.abcam.cn/sorlasorl1-antibody-epr14670-ab190684.html, item number: ab190684), dilution ratio of 1:1000. After the interspinous ligament tissue was isolated, we preserved it by immersing it in formalin solution within 10 minutes. Subsequently, we obtained all 16 immunohistochemical sections with completed staining after laboratory operations such as wax sealing, sectioning, antigen repair, antibody hybridization, color development, and sealing of the tissue. We performed observation under an inverted microscope, and image interceptions were performed separately for the AS and control groups. We assessed the positivity of all immunohistochemical images using Image J software and performed statistical analysis of the positivity of ANXA3 and SORL1 in the AS and control groups separately using IBM SPSS Statistics 25 independent samples t-test.

**Table 4:** Baseline information of the patients used for immunohistochemical analysis.

Patients	Diagnosis	Gender	Age	Height	Weight	BMI	Number of days in hospital (days)	Duration of surgery (min)
N1	Spinal column fracture	Male	39	177cm	53kg	16.9	8	65
N2	Spinal column fracture	Male	44	172cm	66kg	22.3	3	85
N3	Spinal column fracture	Male	38	168cm	72kg	25.5	3	105
AS1	AS combined with kyphoscoliosis	Male	34	144cm	45kg	21.7	11	341
AS2	AS combined with kyphoscoliosis	Male	34	155cm	36kg	14.9	1	320
AS3	AS combined with kyphoscoliosis	Male	43	145cm	65kg	30.9	9	300
AS4	AS combined with kyphoscoliosis	Male	37	174cm	50kg	16.5	9	226
AS5	AS combined with kyphoscoliosis	Male	42	169cm	66kg	23.1	3	78

## Results

### Data Download and Processing, Differential Expression Analysis, Construction of Heat Map and Volcano Map

**Figure 1:**
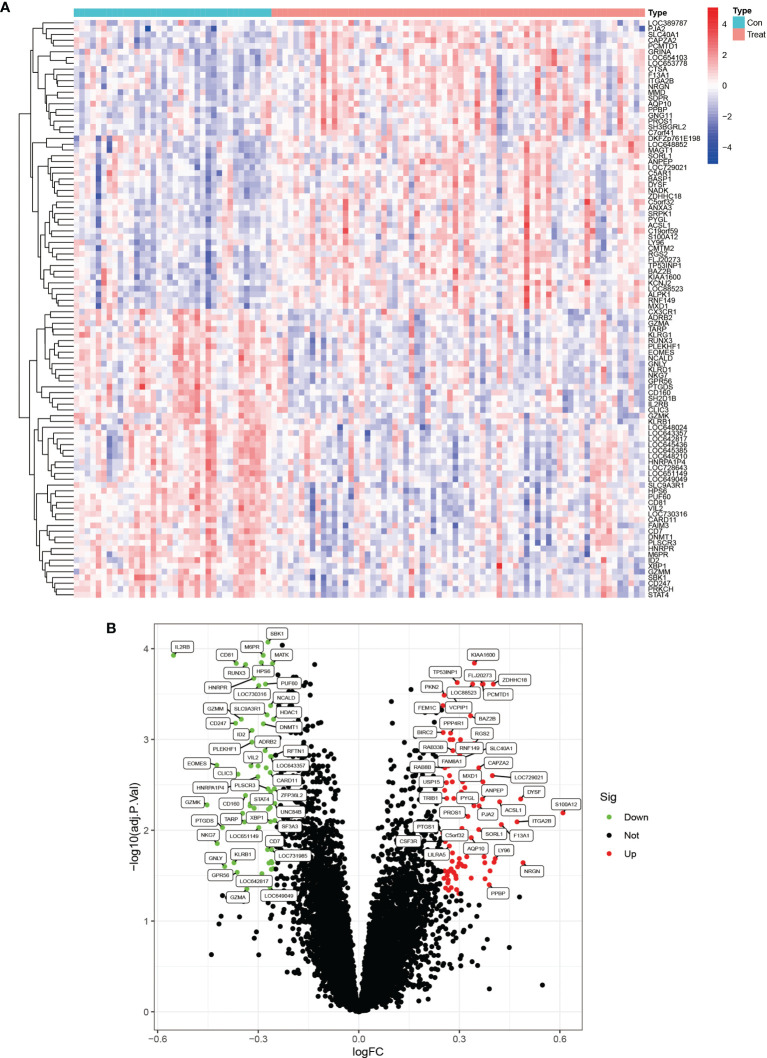
Heat map and volcano plots of differentially expressed genes. **(A)** shows the heat map of differentially expressed genes, red indicates high expression genes and blue indicates low expression genes. **(B)** shows the volcano plots of differentially expressed genes, red is for high expression genes and green is for low expression genes.

We downloaded the two datasets, GSE25101 and GSE73754, from the GEO database that contained a total of 68 AS samples and 36 control samples. After removing inter-batch differences and normalizing the two datasets, we merged the two datasets for analysis. We obtained a list of differentially expressed genes after performing differential expression analysis of gene expressions in AS cases versus control samples and found a total of 295 differentially expressed genes, where the first 100 genes with significant differences were visualized as a heat map, and are shown in **Figure 1A**. The first 50 genes showed higher expression in the AS group than in the control group, while the last 50 genes showed higher expression in the control group than in the AS group. We visualized the differentially expressed genes as a volcano plot (**Figure 1B**), which identified 166 highly expressed genes and 129 low expressing genes.

### GO Enrichment and KEGG Pathway Enrichment Analysis Revealed a Strong Association With Neutrophils and Osteoblasts

**Figure 2:**
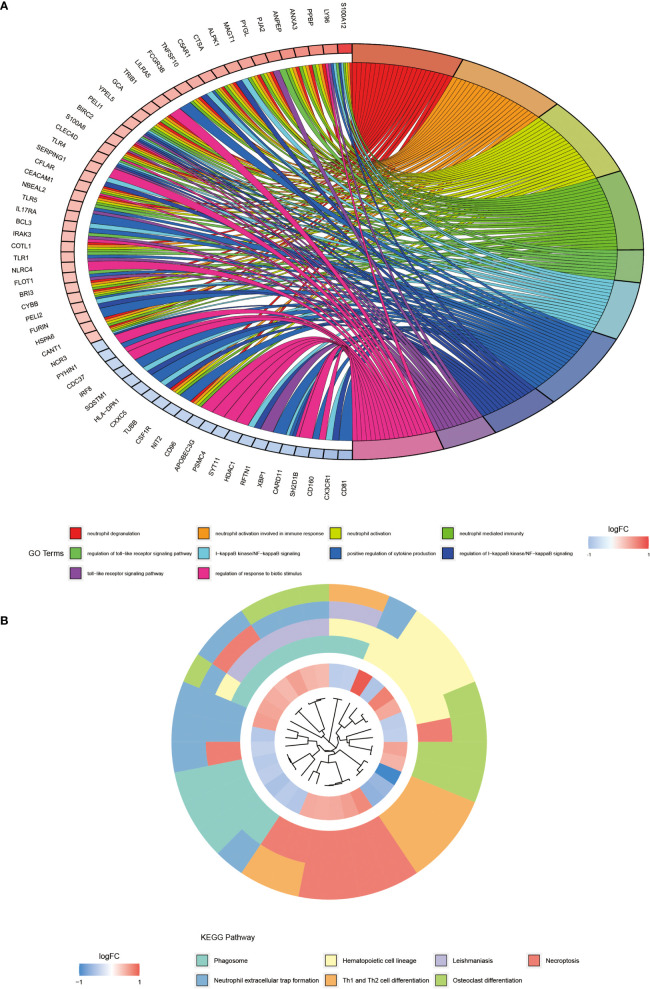
GO and KEGG pathway enrichment analysis of differentially expressed genes. **(A)** shows the first 10 entries of GO enrichment analysis of differentially expressed genes. **(B)** shows the top 7 pathways of KEGG pathway.

We performed the GO enrichment analysis on these differentially expressed genes to analyze their BP, CC, and MF and obtained the results of all enrichment analyses along with visualizing the top 10 most significant ones (**Figure 2A**). **Figure 2A** showed that the GO entries were mainly enriched in neutrophil degranulation, neutrophil activation involved in immune response, neutrophil-mediated immunity, regulation of toll−like receptor signaling pathway, I−kappaB kinase/NF−kappaB signaling, positive regulation of cytokine production, and regulation of response to the biotic stimulus. The KEGG pathway (**Figure 2B**) was mainly enriched in the phagosome, hematopoietic cell lineage, leishmaniasis, necroptosis, neutrophil extracellular trap formation, Th1 and Th2 cell differentiation, and osteoclast differentiation.

### DO and GSEA Analysis Found Close Association With Multiple Inflammatory Diseases

**Figure 3:**
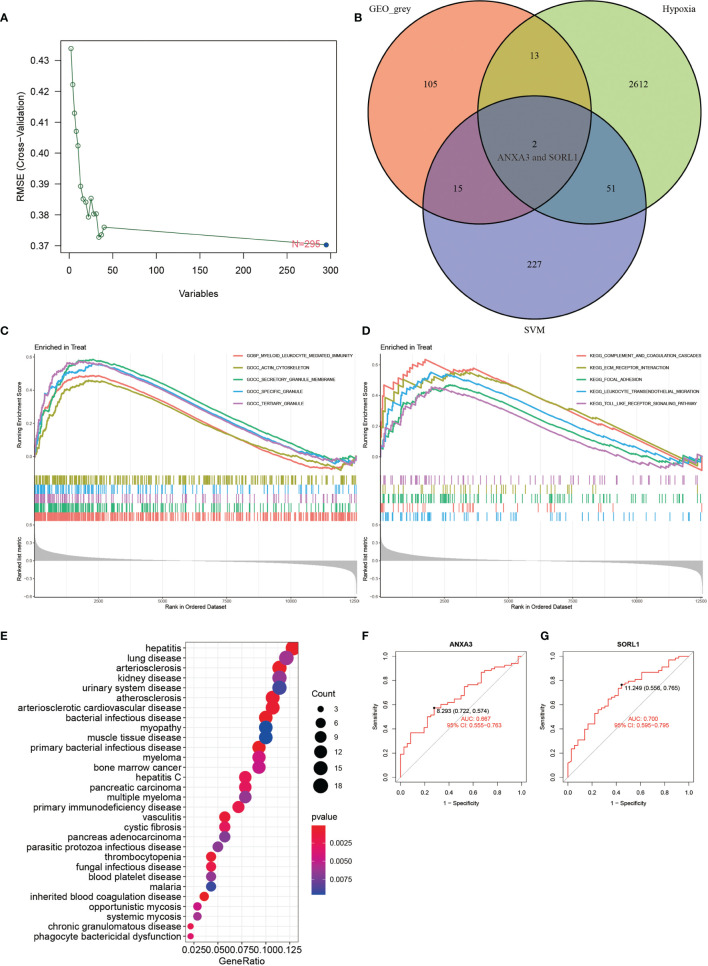
SVM analysis, GSEA, DO analysis, veen plot and ROC curve. **(A)** shows the results of SVM analysis. **(B)** shows the results of intersection of GEO_grey module, hypoxia-related genes and SVM analysis. **(C, D)** show the results of GO and KEGG from GSEA enrichment analysis. **(E)** shows the results of DO enrichment analysis. **(F, G)** show the ROC diagnostic curves of 2 genes of the model. **(F, G)** show the ROC curves of 2 genes of the model.

The result of DO enrichment analysis (**Figure 3E**) showed enrichment mainly in primary immunodeficiency disease, muscle tissue disease, myopathy, phagocytic bactericidal dysfunction, blood platelet disease, cystic fibrosis, and vasculitis. The GSEA enrichment analysis of the AS group showed that the main pathway of GO enrichment was in the myeloid leukocyte-mediated immunity, actin cytoskeleton, secretory granule membrane, specific granule, and tertiary granule (**Figure 3C**), while the KEGG pathway was mainly enriched in the complement and coagulation cascades, ECM-receptor interaction, focal adhesion, leukocyte transendothelial migration, and toll-like receptor signaling pathway (**Figure 3D**).

### ANXA3 and SORL1 Are Genes Used as Diagnostic Models for AS

**Figure 4:**
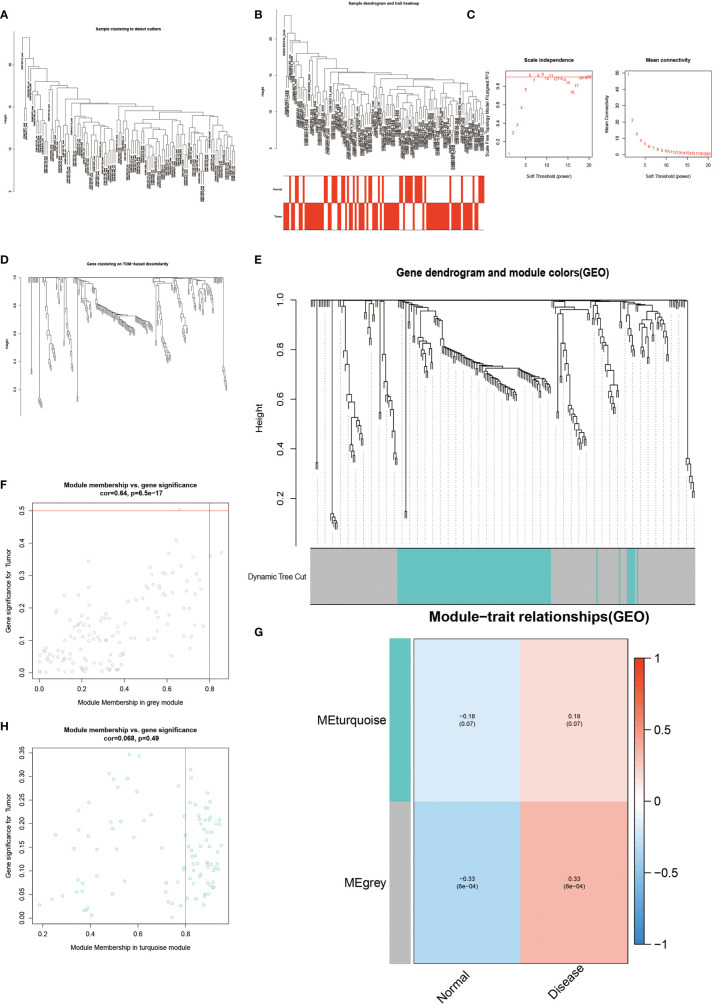
Results of WGCNA analysis. **(A-E)** shows the results of cluster analysis. **(G)** shows the Person correlation with disease and normal groups. **(F, H)** show the scatter plot of correlation.

The results of WGCNA revealed (**Figure 4**) that the MEgrey module showed the highest Pearson correlation coefficient with AS (**Figure 4G**), resulting in a Cor value of 0.33 (p < 0.05). On the other hand, the SVM analysis of all differentially expressed genes showed that a total of 295 genes remained available for diagnosis in the model (**Figure 3A**). All human Hypoxia-associated genes were downloaded from the GSEA database, which contained a total of 207 Hypoxia-associated genes. We then constructed diagnostic models by taking the intersection of genes from the MEgrey module of WGCNA and obtained 295 genes from the SVM analysis along with 2678 Hypoxia-related genes. Our results showed that two genes, ANXA3 and SORL1, met all our screening requirements (**Figure 3B**). Subsequently, we constructed ROC diagnostic curves to test the results of our analysis and found that the area under the curve for the AS diagnostic model constructed by ANXA3 was 0.667 with a 95% CI of 0.558–0.765, specificity is 0.722 and sensitivity is 0.574 (**Figure 3F**). The area under the curve for the AS diagnostic model constructed by SORL1 was 0.700 with a 95% CI of 0.587-0.796, specificity of 0.556 and sensitivity of 0.765 (**Figure 3G**). The area under the ROC curve for both these genes was much greater than 0.5, indicating a more accurate diagnostic curve.

### Relationship Between ANXA3 and SORL1 and Antitumor Drug Sensitivity

**Figure 5:**
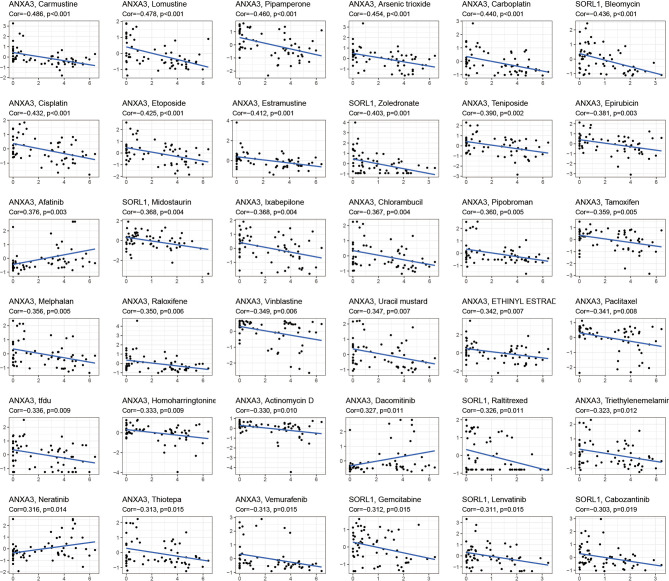
Drug sensitivity analysis. The graph shows the drug sensitivity analysis of ANXA3 and SORL1 with various drugs. If Cor > 0, it means the gene is positively correlated with drug sensitivity, the higher the gene expression, the stronger the sensitivity of this drug; conversely, Cor < 0 means the gene is negatively correlated with drug sensitivity, the higher the gene expression, the worse the sensitivity of this drug.

The drug sensitivity analysis (**Figure 5**) showed that ANXA3 and SORL1 were significantly correlated with the sensitivity of multiple drugs. ANXA3 showed a significant negative correlation (P< 0.05) with several drugs, including Carmustine, Lomustine, Pipamperone, and Arsenic trioxide but had a positive correlation (p < 0.05) with Afatinib, Dacomitinib, and Neratinib. The SORL1 was negatively correlated with Gemcitabine, Lenvatinib, and Cabozantinib (p < 0.05).

### CIBERSORT Analysis Found Close Association With a Variety of Immune Cells Including Neutrophils

**Figure 6:**
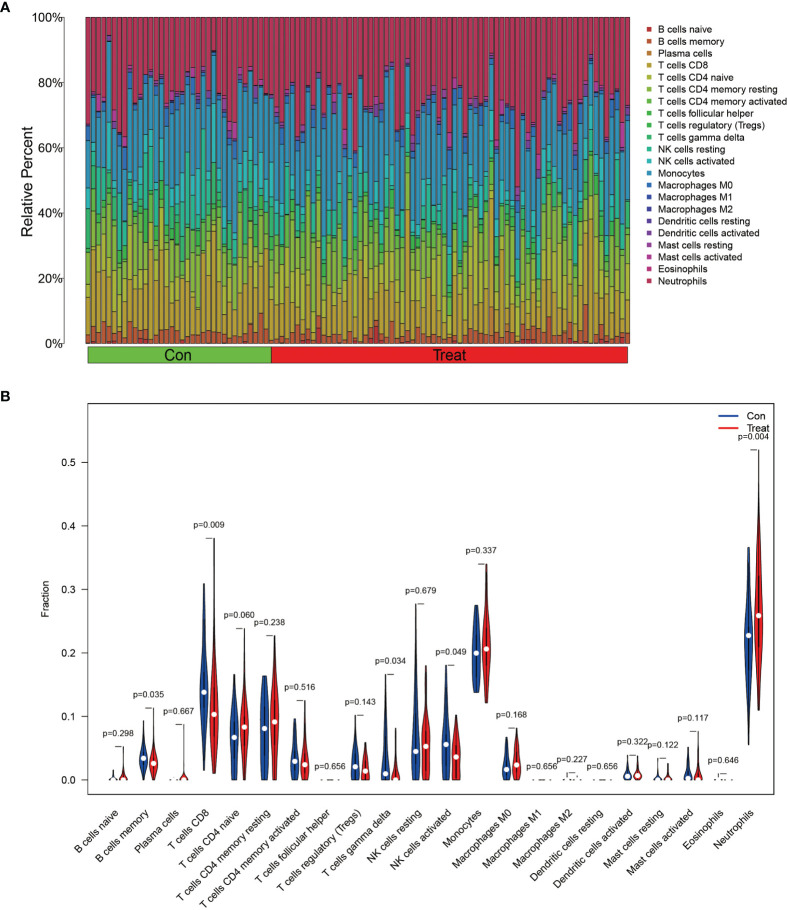
Immune cell composition analysis and differential expression analysis of immune cells. **(A)** shows the immune cell composition of all GEO downloaded samples, each sample consists of 22 immune cells with a total ratio of 100%. **(B)** shows the differences in immune cells in AS and control group, as seen in Neutrophils, NK cells activated, T cells gamma delta, T cells CD8 and B cells memory were significantly different (p < 0.05).

**Figure 7:**
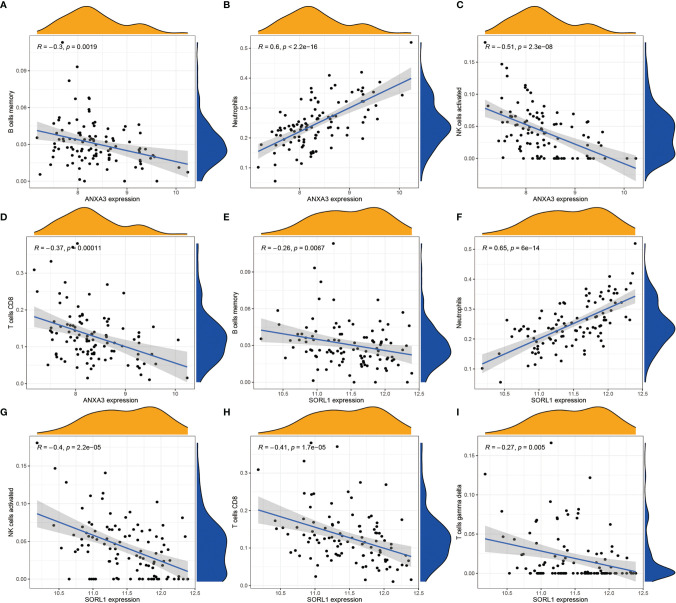
Correlation analysis of ANXA3, SORL1 and immune cells respectively. **(A-D)** shows the correlation analysis of ANXA3 and 4 immune cells. **(E-I)** shows the correlation analysis of SORL1 and 5 immune cells. If R > 0, it means higher gene expression and higher immune cell content; if R < 0, it means higher gene expression and lower immune cell content.

**Figure 8:**
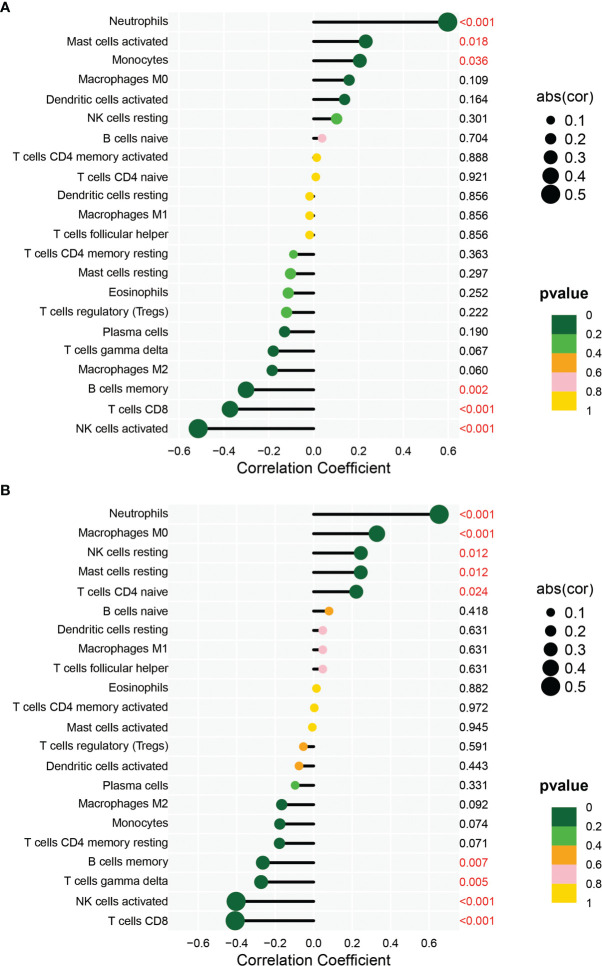
Immune correlation lollipop plot. **(A)** shows the immune cells with significant difference of ANXA3 in AS and control. **(B)** shows the immune cells with significant difference of SORL1 in AS and control.

In order to analyze the composition of immune cells between the AS versus control group, we used CIBERSORT software and obtained the amount of immune cell expression in each sample, which was visualized as a percentage plot (**Figure 6A**). Subsequently, we also analyzed the differences in immune cells between the AS and control groups (**Figure 6B**) and found significant differences in memory B cells, CD8 T cells, activated gamma and delta T cells, NK cells, and neutrophils (P< 0.05). Immediately, we also analyzed the correlation between the two genes used to construct the diagnostic model and the immune cells and then constructed a fitted curve for the correlation analysis between them (**Figure 7**). Interestingly, we found that ANXA3 showed a significant negative correlation with memory B cells and CD8T cells but showed a significant positive correlation with NK cell activation. On the other hand, SOCRL1 showed a significant negative correlation with memory B cells, activated NK cells, and CD8 T cells, while it showed a significant positive correlation with neutrophils. **Figure 8A** showed that six immune cells were correlated with ANXA3 (p < 0.05) while nine immune cells were correlated with SOCRL1 (p < 0.05, **Figure 8B**).

### High-Throughput Sequencing and Routine Blood Test Results

**Figure 9:**
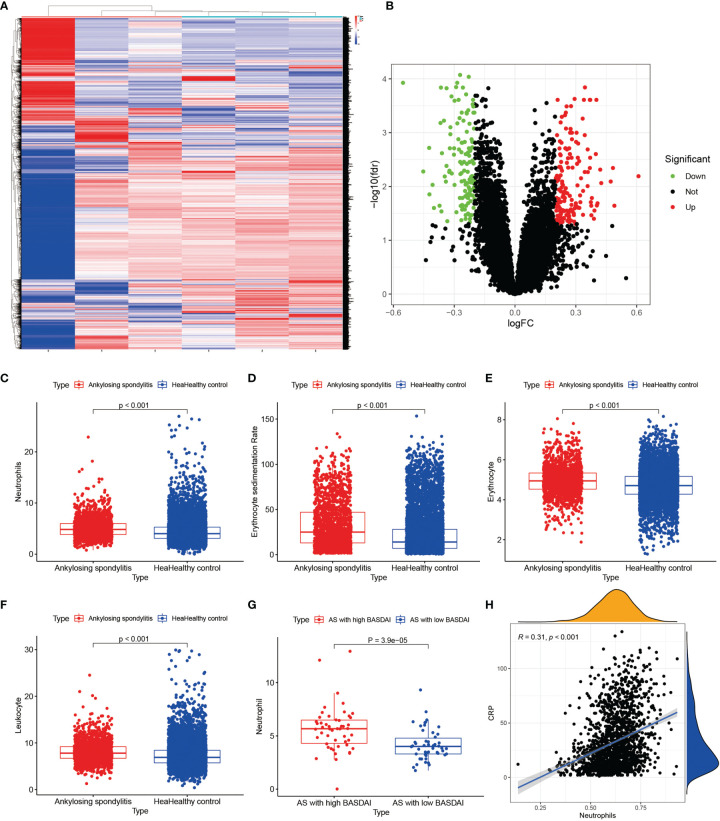
Results of external validation sets are shown. **(A, B)** show heat and volcano maps of differentially expressed genes measured by high-throughput sequencing, confirming that ANXA3 and SORL1 are also differentially expressed genes in the interspinous ligament of AS. **(C-F)** show the variance analysis of the routine blood tests. **(G)** shows the difference in neutrophils between high BASDAI cases and low BASDAI cases. **(H)** shows the relationship between neutrophils and CRP.

We performed RNA extraction from the interspinous ligaments of three AS patients and three lumbar degenerative interspinous ligament cases who met the inclusion-exclusion criteria. We finally obtained the gene expression of mRNAs of all AS and normal controls by the above mentioned laboratory manipulations. The differential expression analysis on the validation set was based on the cut-off values of the experimental set, using which, we obtained all the differentially expressed genes. Then, we constructed the heat maps and volcano maps of differentially expressed genes (**Figures 9A, B**) and found that the difference between ANXA3 and SORL1 in the validation set was highly significant. Thus, we further tested the accuracy of our bioinformatics analysis. Also, we compared the neutrophils (**Figure 9C**), Erythrocyte sedimentation Rate (**Figure 9D**), red blood cells (**Figure 9E**), and white blood cells (**Figure 9F**) between 1770 HLA-B27-positive cases and 7939 HLA-B27-negative cases and found that the values of these indicators were significantly higher in AS than in the healthy controls (P<0.001). Our statistical analysis by the number of neutrophils with AS with high BASDAI scores versus AS with low BASDAI scores revealed that the number of neutrophils with AS with high BASDAI scores was significantly higher than the number of neutrophils with low scores (**Figure 9G**), and the difference was statistically significant (p < 0.001). On the other hand, the correlation analysis of neutrophils and CRP (**Figure 9H**) showed a positive correlation between neutrophils and CRP with a correlation coefficient of 0.31, a statistically significant difference (p < 0.001).

### Immunohistochemical Analysis Results

**Figure 10:**
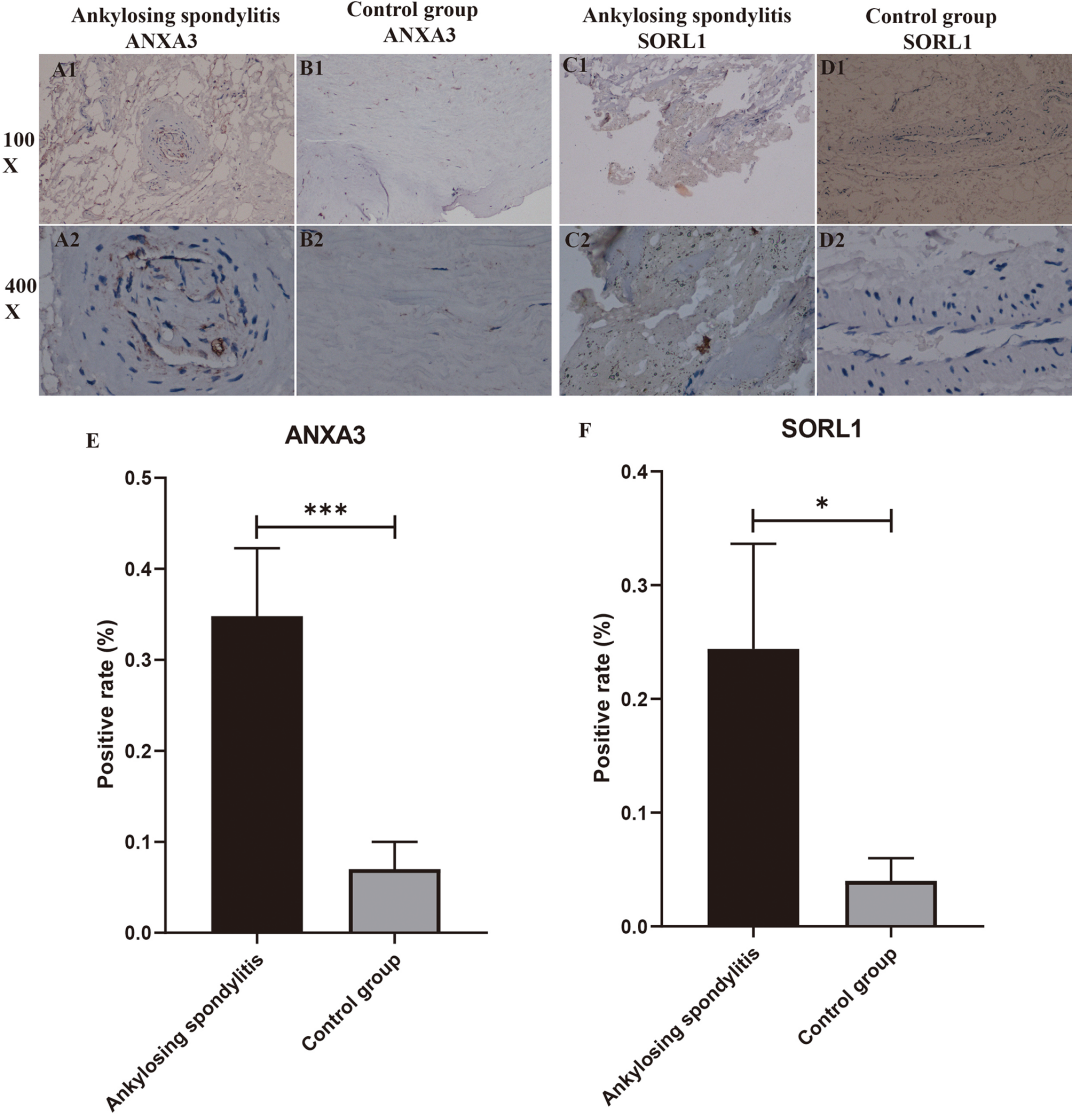
Immunohistochemistry and positive rate statistics. **(A1-D2)** show the specific expression of ANXA3 with SORL1 in AS and controls. **(E, F)** shows the positive rate statistics of all immunohistochemical images of SORL1 in AS and control groups. “*” indicates p < 0.05, “***” indicates p < 0.001.

We performed immunohistochemical staining of the interspinous ligaments of five patients with AS and three patients with spinal fractures for ANXA3 and SORL1, respectively. The results revealed that the specific expression of each of ANXA3 and SORL1 in AS was significantly higher than that in the control group (**Figures 10A1-D2**). After detecting the positive rate of all immunohistochemical images with Image J software, the positive rate data of ANXA3 and SORL1 were imported into IBM SPSS Statistics 25 software and statistically analyzed by independent sample t-test, and the positive rate statistics of ANXA3 and SORL1 were found to be much higher in AS than in the control group (**Figures 10E, F**), and the difference was statistically significant (P<0.05). This further confirms the accuracy of our preliminary analysis, demonstrating that ANXA3 and SORL1 are differentially expressed in AS and control groups.

## Discussion

In our study, we conducted GO enrichment analysis of 68 AS samples and 36 control samples obtained from the GEO database for differentially expressed genes, and our results showed that entries were mainly enriched in neutrophil degranulation, neutrophil activation involved in immune response, and neutrophil-mediated immunity. Previous studies have shown that neutrophils in cells have a limited pro-inflammatory function (35). Reactive oxygen species (ROS) have also been an important factor in the progression of inflammatory diseases. The enhanced ROS production by polymorphonuclear neutrophils in inflammation can cause tissue damage and endothelial dysfunction. Also, the oxidative stress produced by neutrophils in an inflammatory environment leads to the opening of endothelial junctions, facilitating the passage of inflammatory cells across the endothelial barrier (36). Neutrophils are the immune cells involved in the development of many diseases. Interestingly, our diagnostic model showed that both ANXA3 and SORL1 showed a significant positive correlation with neutrophils (p < 0.05) with neutrophil expression being much higher in the AS patients than in the controls (p < 0.01). Enrichment analysis of the KEGG pathway showed that it was mainly enriched in neutrophil extracellular trap formation, Th1 and Th2 cell differentiation, and osteoclast differentiation. Fernanda V S Castanheira et al. showed that neutrophils can fight inflammation through phagocytosis and/or by releasing extra-neutrophilic traps (NETs). The neutrophils also help in repairing already damaged tissues while limiting the production of NETs (37). This was also in line with our study, which showed that a significant rise in neutrophils might contribute to the progression of AS.

Annexin A3 (ANXA3) is a protein-coding gene, which is associated with diseases that mainly include ovarian cancer and upper urinary tract uroepithelial carcinoma. Previous studies have shown that miR-18b can prevent cerebral ischemia-reperfusion injury by activating the PI3K/Akt pathway and inhibiting ANXA3 in an oxygen-glucose deprivation/reperfusion model (OGDR) *in vitro* and middle cerebral artery occlusion model (MCAO) *in vivo* simulating the cerebral ischemia-reperfusion injury (38). Also, ANXA3 has been identified as a potential biomarker in Zhizhong Yan et al. ‘s study of aneurysmal subarachnoid hemorrhage, which was identified through the construction of a coexpression network by WGCNA (39). However, to date, no studies have reported ANXA3 in AS, and our study is the first to suggest that dysregulation of ANXA3 may contribute to the progression of AS and lead to dysregulation of immune cells, particularly neutrophils, thereby contributing to the development of the disease. Our high-throughput sequencing results showed that ANXA3 is also a significantly different gene found in the interspinous ligaments of AS. On the other hand, we compared the neutrophil counts of 1770 HLA-B27-positive cases and 7939 HLA-B27-negative cases and found that the neutrophil counts in the AS group were significantly higher than those of the healthy controls, further confirming our bioinformatics analysis. On the other hand, the results of immunohistochemical analysis of ANXA3, which we performed in order to test *in vitro* whether there were differences between AS and normal groups, also showed that its specific expression was significantly higher in AS than in normal controls, with statistically significant differences (p < 0.05). This result is consistent with the results of our analysis.

Sortilin Related Receptor 1 (SORL1) is a protein-coding gene that is currently known to be most closely associated with Alzheimer’s disease (40–42). Abnormalities within the neurons in the lysosomal and autophagy/macroautophagy systems are prominent features of the early stages of Alzheimer’s disease (AD). SORL1 mutations are an important cause of rare autosomal dominant AD, increasing the risk of late-onset AD (43). The deletion of SORL1 may affect the regulatory protein-induced cell proliferation enabling metastatic resistance to HER2 treatment in breast cancer cells (44). Interestingly, our KEGG pathway enrichment analysis showed that it was mainly enriched in the phagosome, neutrophil extracellular trap formation, and osteoclast differentiation pathways. Interestingly, our findings suggested that SORL1 is a key differentially expressed gene in AS. Also, our results from high-throughput sequencing showed that it is an important differentially expressed gene in the pathogenesis of AS. Moreover, SORL1 showed a significant positive correlation with neutrophil expression (Cor = 0.65, p < 0.001), where the neutrophil counts were significantly higher in AS than that of the healthy controls. We also obtained the data of neutrophils from routine blood tests from 1770 HLA-B27-positive cases and 7939 HLA-B27-negative cases. Since no reports have identified and studied SORL1 in AS, we propose for the first time that it may also be a possible key gene in the pathogenesis of AS. We found that SORL1 expression was significantly higher in AS than in normal controls by immunohistochemistry of SORL1 in the interspinous ligaments of five AS cases and in the interspinous ligaments of three spinal fractures, and the difference was statistically significant (P < 0.05). This result tests the accuracy of our analysis at the level of *in vitro* experiments. In our study, we found that both ANXA3 and SORL1 showed a significant positive correlation with neutrophil count. Thus, in order to assess the relationship between ANXA3 and SORL1 and disease activity, we took the neutrophil counts of the first 50 patients with BASDAI scores above the mean and the last 50 patients with BASDAI scores below the mean for statistical analysis, and we found that the neutrophil counts of patients with high scores were significantly higher than those of patients with low scores, and the differences were statistically significant (p < 0.001). This result indirectly confirms a strong association of ANXA3 and SORL1 with disease progression in AS patients.

We first screened the differentially expressed genes in AS by combining the two GEO datasets and then performed GO enrichment analysis, KEGG pathway enrichment analysis, DO enrichment analysis, GSEA enrichment analysis, followed by SVM analysis, WGCNA, and intersection with Hypoxia-related genes to obtain the diagnostic models for the genes ANXA3 and SORL1. We also performed a drug sensitivity analysis, immune cell content analysis, and immune cell differences analysis and found that both ANXA3 and SORL1 showed a significant positive correlation with neutrophils, which was confirmed by the routine blood data obtained from AS and healthy control samples. Also, we confirmed that these two genes were indeed differentially expressed in AS by high-throughput sequencing of three AS samples and three lumbar degeneration samples. We also analyzed the staining by immunohistochemistry for ANXA3 and SORL1, respectively, whose results revealed that both ANXA3 and SORL1 were more expressed in AS than in normal controls. The results of immunohistochemistry also supported the results of our analysis. We analyzed and tested for differences in immune cells using a total of 9707 cases of routine blood data, and the test results were consistent with our analysis.

Similar to other studies, our study also had limitations. Firstly, the sample size was inadequate. Our bioinformatics analysis used a total of 104 samples, including 68 AS samples and 36 control samples, while three AS interspinous ligament samples and three Degenerative lumbar spine samples were used for high-throughput sequencing. Also, we collected data on neutrophil counts from a total of 361 cases belonging to 1770 HLA-B27-positive cases and 7939 HLA-B27-negative cases, which is far from adequate for a large sample size. Secondly, we did not have more laboratory analyses to test our results and used only two different external validations, which is not nearly enough.

## Conclusion

Upregulated of ANXA3, SORL1, and neutrophils can be key factors in the progression of Ankylosing Spondylitis.

## Data Availability Statement

The datasets supporting the conclusions of this article are available in the GEO database: https://www.ncbi.nlm.nih.gov/geo/query/acc.cgi?acc=GSE25101 and https://www.ncbi.nlm.nih.gov/geo/query/acc.cgi?acc=GSE73754.

## Ethics Statement

The studies involving human participants were reviewed and approved by Ethics Department of the First Affiliated Hospital of Guangxi Medical University. Written informed consent for participation was not required for this study in accordance with the national legislation and the institutional requirements.

## Author Contributions

JJ, CL, and XZ designed the study. TL, LC, SH, and XS analyze the data. WJ, JC, TC, HL, and YY digital visualization. SW and JZ collected data on routine blood data. JJ, HQ, HL, TL, YY, XZ, and CL performed the laboratory operation. JJ wrote and revised the manuscript. CL and XZ revised the manuscript. All co-authors participated in the laboratory operation. All authors contributed to the article and approved the submitted version.

## Funding

This study was supported by the Youth Science Foundation of Guangxi Medical University, Grant/Award Numbers: GXMUYFY201712, Guangxi Young and Middle-aged Teacher’s Basic Ability Promoting Project, Grant/Award Number: 2019KY0119.

## Conflict of Interest

The authors declare that the research was conducted in the absence of any commercial or financial relationships that could be construed as a potential conflict of interest.

## Publisher’s Note

All claims expressed in this article are solely those of the authors and do not necessarily represent those of their affiliated organizations, or those of the publisher, the editors and the reviewers. Any product that may be evaluated in this article, or claim that may be made by its manufacturer, is not guaranteed or endorsed by the publisher.
